# Supplementary figures and images for: Preformation and epigenesis converge to specify primordial germ cell fate in the early Drosophila embryo
Source: PLoS Genet. 2022 Jan 5;18(1):e1010002. doi: 10.1371/journal.pgen.1010002 (PMC8765614; doi:10.1371/journal.pgen.1010002)

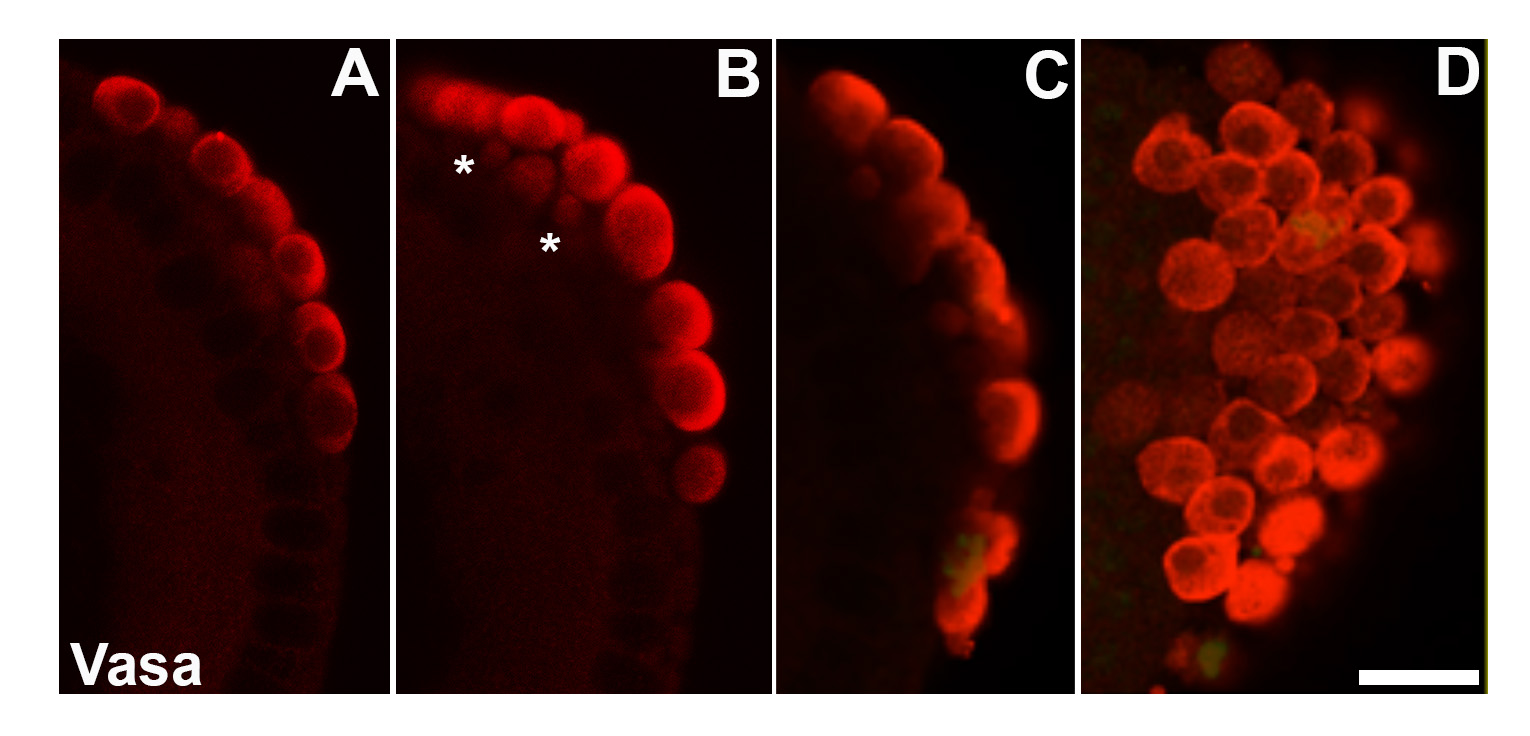

Supplement: S1 Fig — 0–4 hr paraformaldehyde-fixed (A/C) twi-Gal4 (B/D) or twi-Gal4/UAS-dpp stage 4 (A-B) or stage 5 (C-D) embryos were stained for pole cell marker Vasa (red) to assess pole cell number and proliferation. Asterisks indicate additional divisions. Scale bar represents 10 μm. (JPG) [file pgen.1010002.s001.jpg]

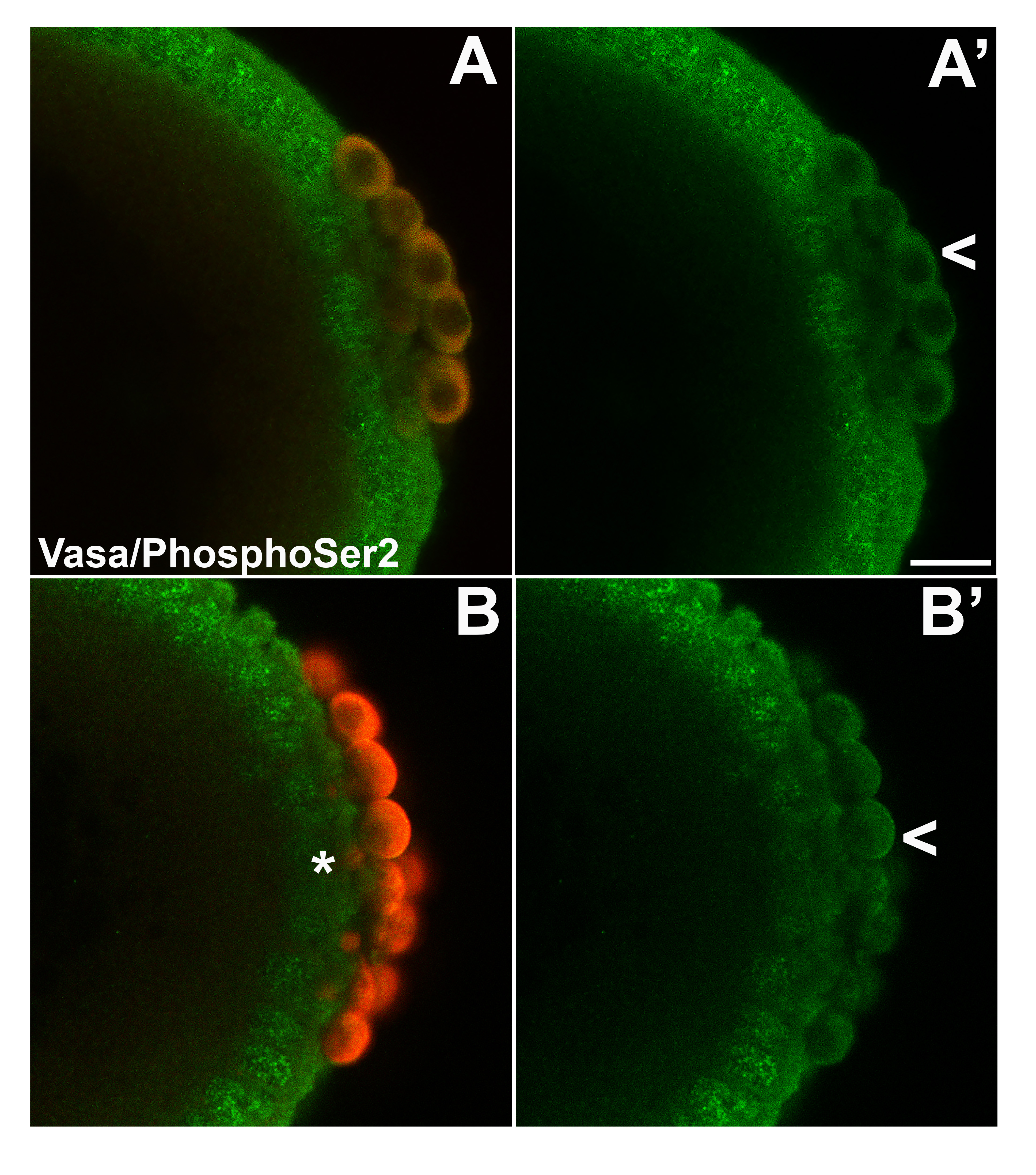

Supplement: S2 Fig — 0–4 hr paraformaldehyde-fixed (A) twi-Gal4 (B) or twi-Gal4/UAS-dpp embryos were stained for pole cell marker Vasa (red) and phosphoSer2 (transcriptional activation, green). Asterisk indicates excess division shown by greater number of Vasa-positive cells. Carets shows lack of pSer2 in PGCs, suggesting transcriptional quiescence. Scale bar represents 10 μm. (JPG) [file pgen.1010002.s002.jpg]

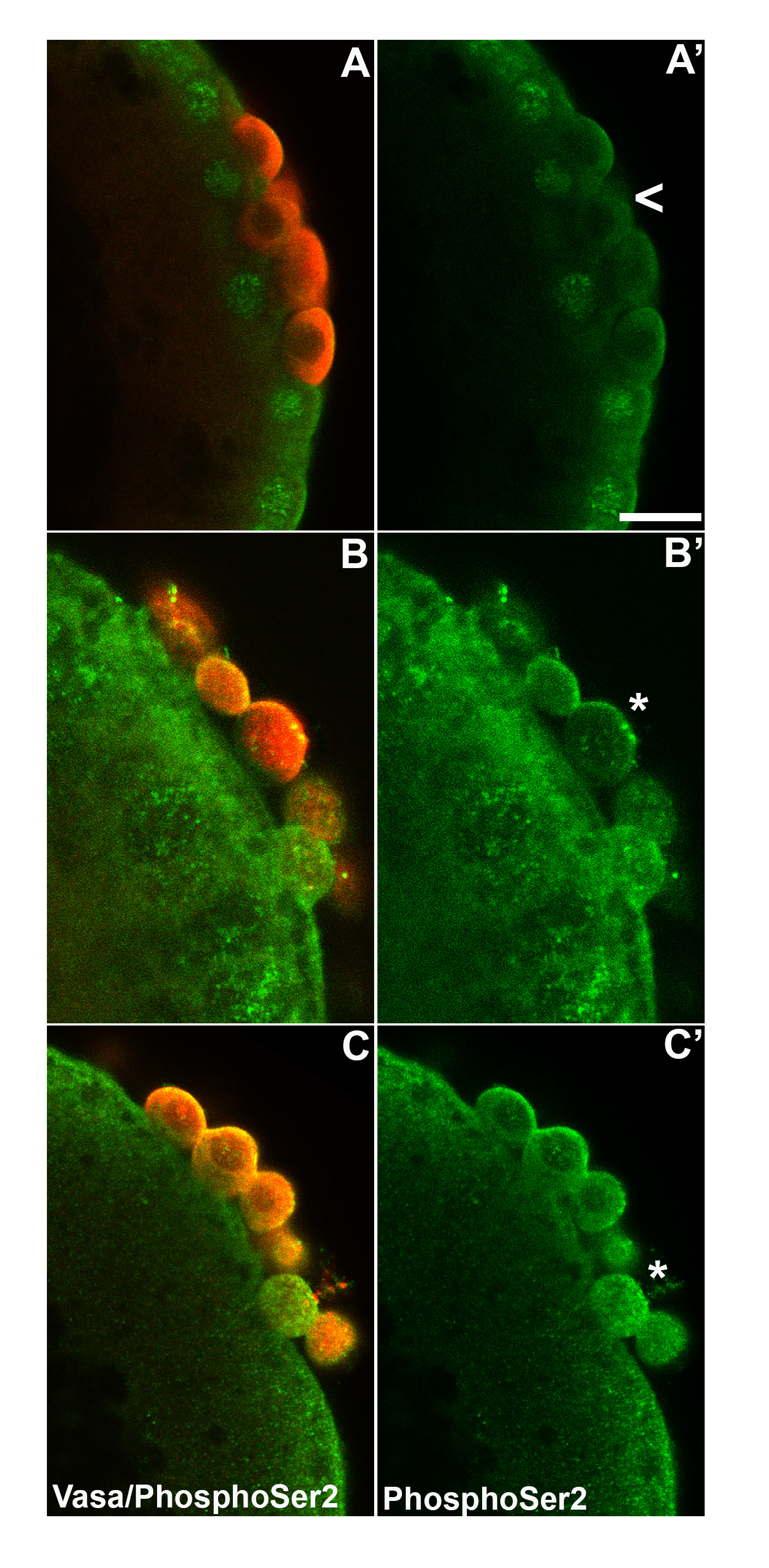

Supplement: S3 Fig — 0–4 hr paraformaldehyde-fixed (A) WT and (B/C) tkvm- embryos were stained for pole cell marker Vasa (red) and phosphoSer2 (transcriptional activation, green). Caret highlights absence of pSer2 signal, suggesting transcriptional quiescence, while asterisk shows that loss of Vasa correlates with gain of transcriptional activation. Scale bar represents 10 μm. (JPG) [file pgen.1010002.s003.jpg]

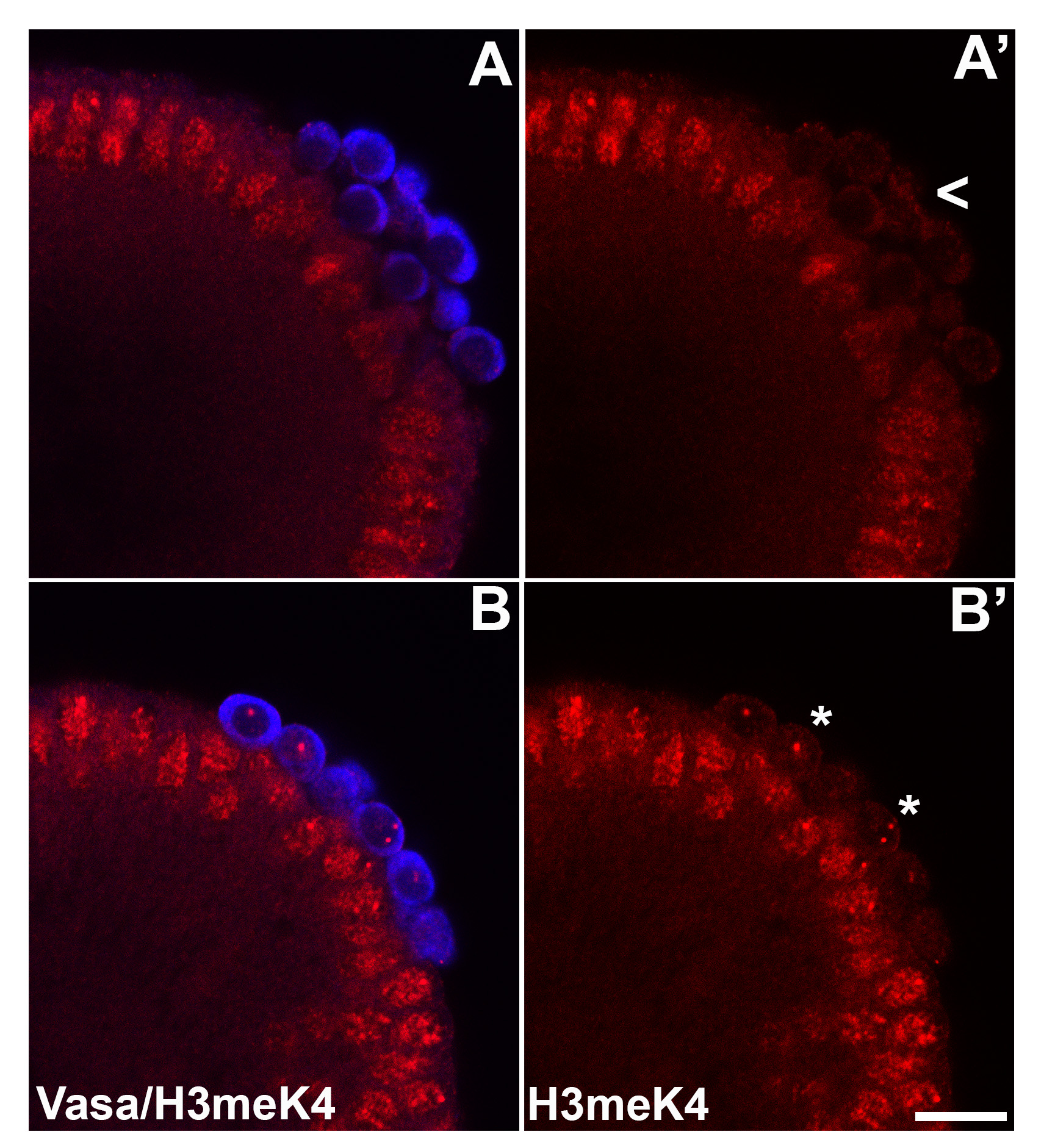

Supplement: S4 Fig — 0–4 hr paraformaldehyde-fixed (A) WT and (B) tkv421 embryos were stained for the pole cell marker Vasa (blue) and H3meK4 (red). Caret shows absence of H3meK4 while asterisk highlights aberrant presence of H3meK4 signal in PGCs, a marker of transcriptionally active chromatin. Scale bar represents 10 μm. (JPG) [file pgen.1010002.s004.jpg]

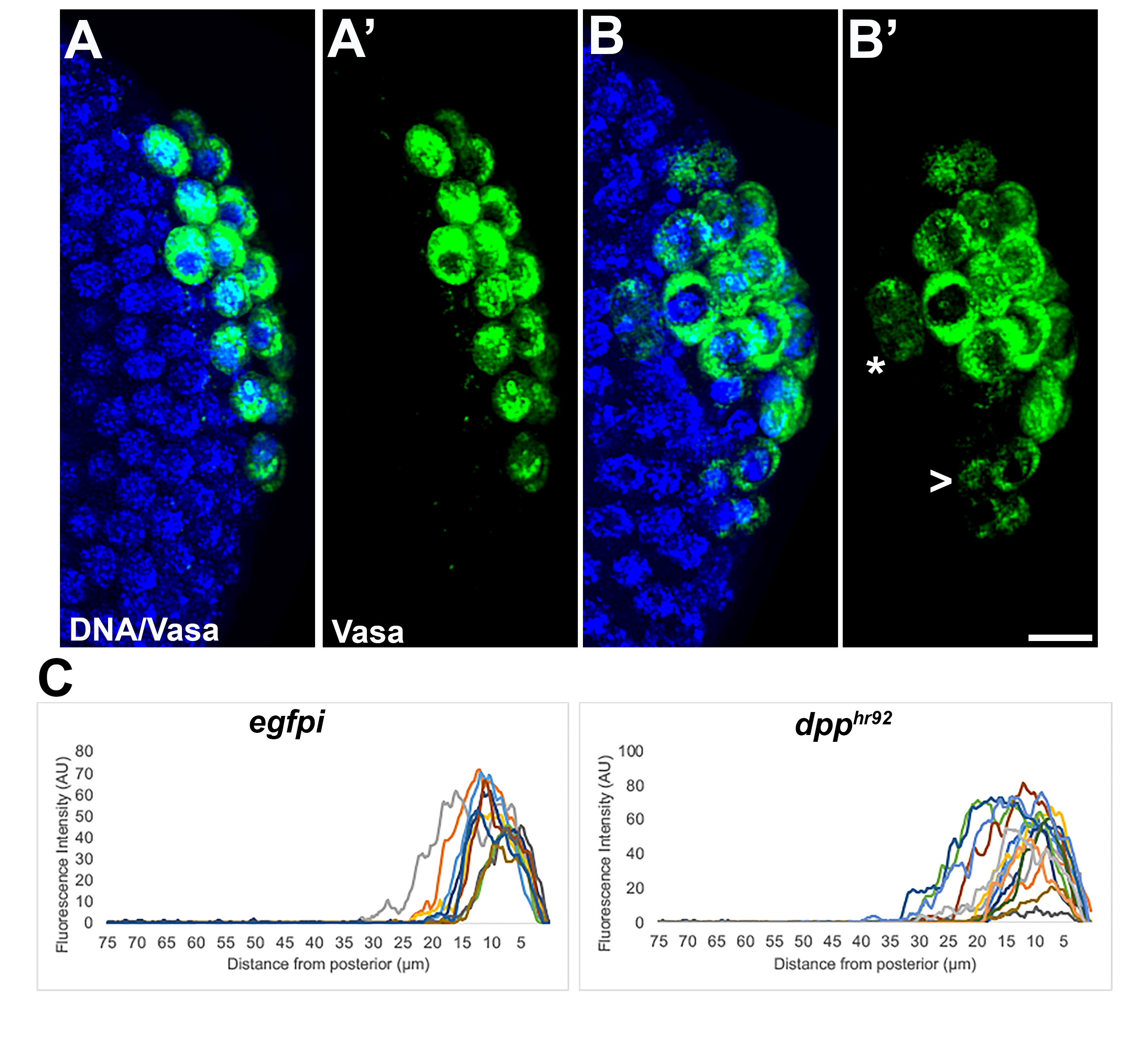

Supplement: S5 Fig — 0–4 hr paraformaldehyde-fixed (A) egfpi and (B) dpphr92 embryos were stained for pole cell marker Vasa (green). Nuclei were labeled using Hoescht (blue). Asterisk shows Vasa localization away from posterior pole and invasive migration of PGCs. Scale bar represents 10 μm. (C) Plot profiles showing mislocalization of pole plasm (visualized using Vasa) away from posterior cap (see Materials and Methods for details of quantification). (JPG) [file pgen.1010002.s005.jpg]

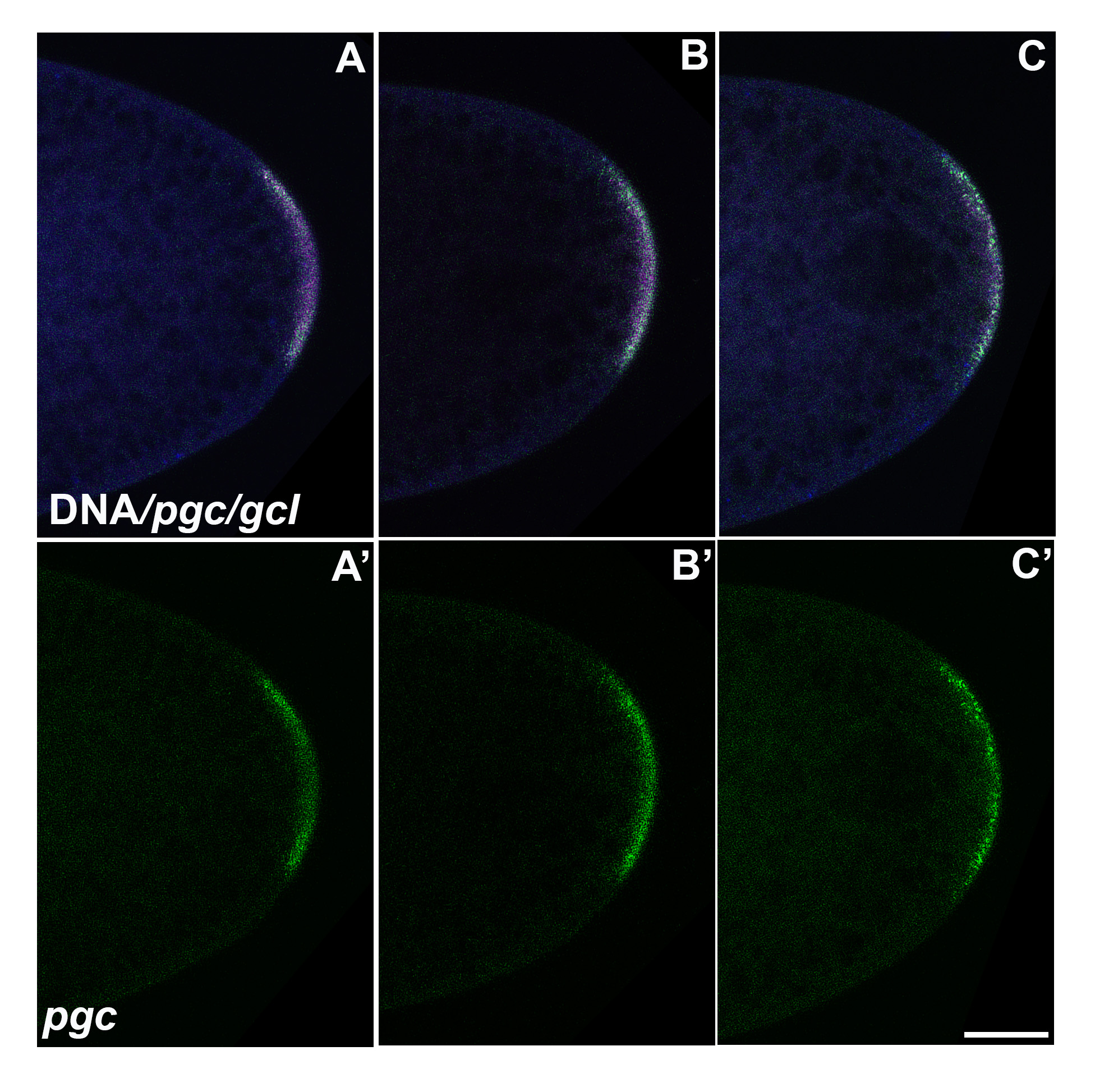

Supplement: S6 Fig — smFISH was performed using probes specific for pgc (green) and gcl (magenta) on 0–4 hr paraformaldehyde-fixed (A) egfpi (B) dpphr92 and (C) dppi33618 embryos. (JPG) [file pgen.1010002.s006.jpg]

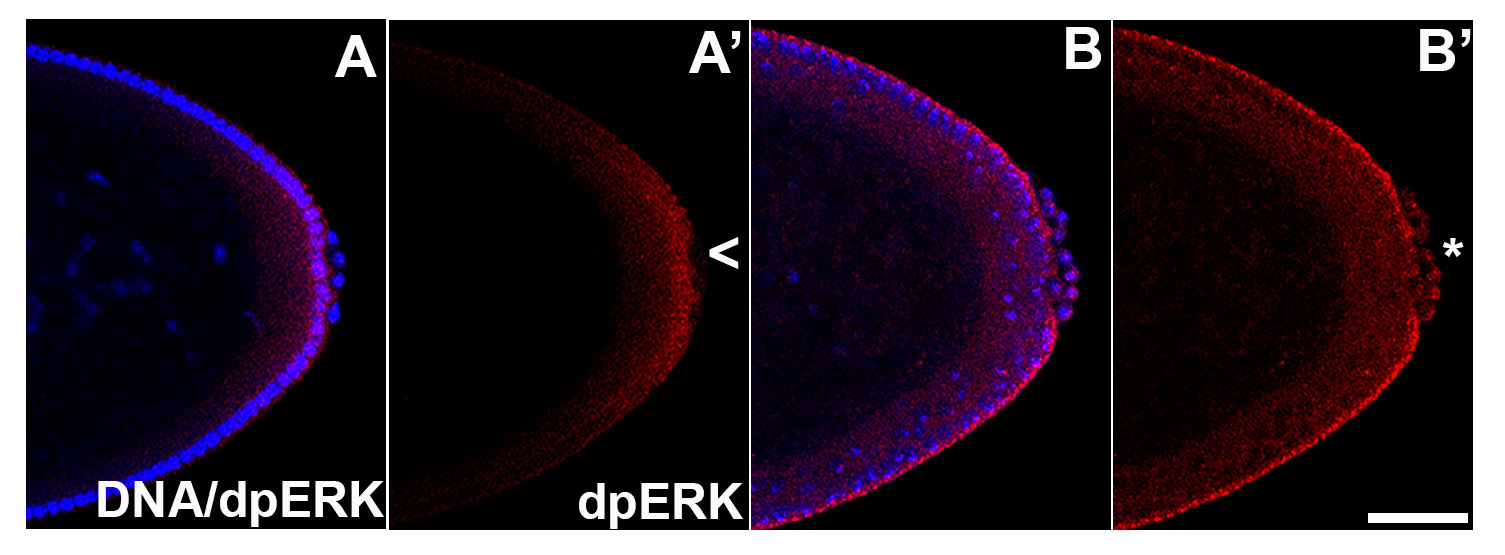

Supplement: S7 Fig — 0–4 hr paraformaldehyde-fixed (A) WT and (B) dpphr92 embryos were stained for dpERK (red). Nuclei were labeled using Hoescht (blue). Caret indicates lack of dpERK in WT PGCs while the asterisk highlights ectopic dpERK in dpphr92 PGCs. Scale bar represents 10 μm. (JPG) [file pgen.1010002.s007.jpg]

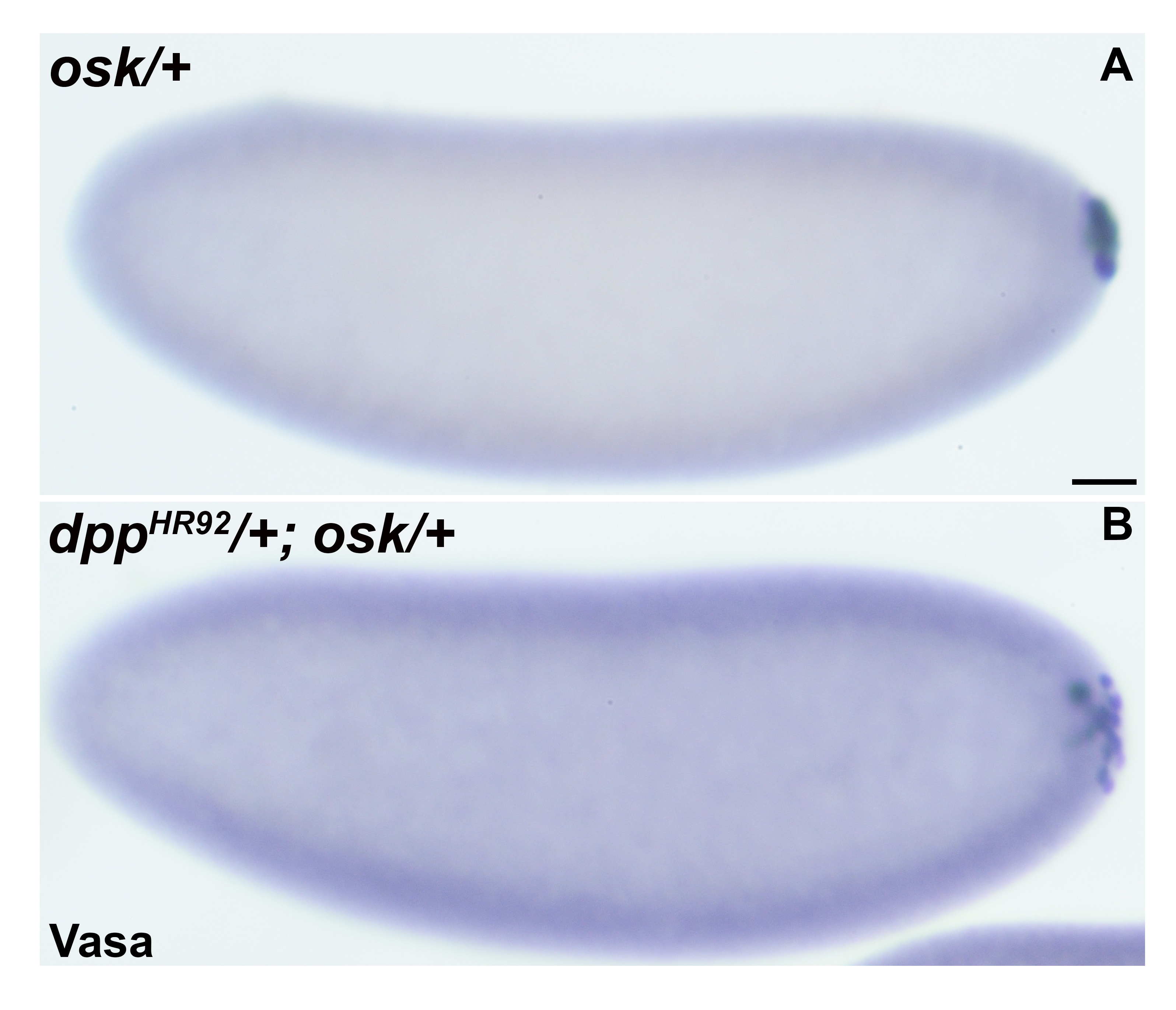

Supplement: S8 Fig — 0–4 hr paraformaldehyde-fixed (A) osk/+ and (B) dpphr92/+;osk/+ embryos were stained for the pole cell marker Vasa. Scale bar represents 10 μm. (JPG) [file pgen.1010002.s008.jpg]
