# Supplementary material for: Preformation and epigenesis converge to specify primordial germ cell fate in the early Drosophila embryo
Source: PLoS Genet. 2022 Jan 5;18(1):e1010002. doi: 10.1371/journal.pgen.1010002 (PMC8765614; doi:10.1371/journal.pgen.1010002)
Supplement: Attachment — Submitted filename: Deshpande PLOSG 21.docx [file pgen.1010002.s019.docx]

This is a beautifully crafted and conceptualized study of germ cell specification that is an important new milestone in our understanding of the germ line development. My suggestions are minor. Strong and convincing evidence is provided to show that Dpp has a critical role at syncytial blastoderm stages and mention is made that Dpp is also important at later embryo stages as well, but no explicit statement or model is offered to suggest where the Dpp sources might be. Perhaps the authors could describe their thinking? My other comments are the small details described in the specific suggestions that follow.

Abstract

Ln23	Confusing as not clear if “deployed refers to in a particular animal or, as apparently intended, in biology.

Ln 24	is a signal is a pathway

Ln 28	not clear if “longstanding model” refers to epigenesis in mammals or preformation in Drosophila

Author Summary

Ln 37	“to insulate newly formed PGCs from the adverse effects of the cell-cell signaling pathways” - is this an assumption or is it really proven to be the one and only reason for attenuating the cell cycle?

Introduction

Ln 63	what is a “somatic” gene?

Ln 65	Because all the lineages are fixed in worms, is it correct to distinguish one product of the first division as “set aside”?

Ln 69	What cycle is this minor wave of ZGA?

Ln 71	What cycle is this major wave of ZGA?

Ln 85	Not clear what the new term “model invertebrates” refers to – flies and worms? Does “higher animals” refer to mouse and human, or to other vertebrates as well?

Results

Ln 172	The described result implies that twi-Gal4 is expressed and effects expression of UAS targets at stage 5/6. It would be reassuring to examine expression of a UAS target directly, perhaps UAS-GFP?

Fig 1	Please show twiGal4 expression at the stages analyzed for Cyclin B, pH3, and PGC number.

Ln 216	Perhaps change “significantly” to “detectably”?

Ln 276	Change “embryos” to “embryo”

Ln 408	Please explain why dpERK was monitored

Ln 513	Adhesion is assumed, not demonstrated by these observations of distribution/localization

M&M

Ln 652	Michael O’Connor mis-spelled
