# Supplementary material for: Preformation and epigenesis converge to specify primordial germ cell fate in the early Drosophila embryo
Source: PLoS Genet. 2022 Jan 5;18(1):e1010002. doi: 10.1371/journal.pgen.1010002 (PMC8765614; doi:10.1371/journal.pgen.1010002)
Supplement: Attachment — Submitted filename: Colonnetta et al._PLOS Genetics Response_Final.docx [file pgen.1010002.s020.docx]

Gregory P. Copenhaver
Editor-in-Chief
PLOS Genetics

Dear Dr. Copenhaver,

Dear Dr. Bosco,

Thank you for the reviews of our manuscript (PGENETICS-D-21-01030: Preformation and epigenesis converge to specify primordial germ cell fate in the early *Drosophila* embryo).

We were happy to note that the reviewers were intrigued by the proposed role for epigenesis in PGC development and are grateful for their very useful comments.

In our reading of the reviews, referee #2 appeared to be in favor of publication (with a few minor revisions), while referees #1 and #3 expressed concerns regarding the precise role of BMP signaling in fate specification. In the case of referee #1, a major concern was when and where *dpp* is expressed in the early *Drosophila* embryo. They indicated that the *dpp* expression domain likely did not encompass the PGCs and thus could not logically impact their specification. In addition, they also suggested that *dpp* expression likely commenced after PGC formation and specification. As detailed further below, both published and our unpublished experiments indicate that the *dpp* expression domain fully encompasses the PGCs, and that expression commences prior to PCG cellularization and continues throughout the period of time that we examine in this manuscript. Thus, PGCs are likely bathed in the Dpp ligand from the time they begin to cellularize until the end of syncytial blastoderm stage. Referee #3 also raised questions about the domain and timing of *dpp* expression. Also, like referee #1, referee #3 questioned our use of the word specification. In our response, we have attempted to justify the use of this term (or related terms –fate, identity) with respect to the functions of the BMP signaling pathway and functions of the localized maternal determinants. We hope that our response as well as rewording of different sections of the manuscript will meet with the approval of referees #1 and #3.

In closing, we have modified the text to address the concerns raised by all the reviewers, most notably several of the issues raised by reviewer #1 and #3. Please find a detailed response to the referee’s comments below. We hope that the revised version of the manuscript will be considered suitable for publication in *PLOS Genetics*.

Sincerely,

Girish Deshpande

**Responses to Reviewer Comments:**

Comments to the Authors:

Please note here if the review is uploaded as an attachment.

**Reviewer #1:** In this paper, Colonetta et al suggest that Dpp signalling contributes to germ cell formation in Drosophila and frame this suggestion in the context of the classic epigenesis/preformation debate. The authors show that the number of germ cells is somewhat modulated by changes in Dpp signalling (LOF and GOF). They show that this could be mediated in part by an effect on PGC proliferation but then argue, based on changes in vasa expression, that Dpp regulates an early step in PGC specification. They then describe a number of observations suggesting that the PGC state is ‘weakened’ upon loss of Dpp activity.

The paper starts with an intriguing idea, that germ cells specification relies on a combination of epigenesis and cytoplasmic determinants but unfortunately the evidence is somewhat incomplete, dampening initial enthusiasm. In light of the unexpected nature of their finding, there is a particular burden on proof placed on the authors.

Here are some of the issues (both logical and experimental).

1) The author suggest that Dpp could contribute to the specification of PGCs. However, Dpp expression begins after PGCs have budded off (and hence have been specified). It conceivable that Dpp signalling contributes to maintenance of the PGC fate, but this is not how the authors frame their observations.

“Specification” isn’t necessarily complete when the PGCs have budded off. PGC budding and cellularization is controlled (at least in part) by *gcl*, while other key *osk* dependent “specification” factors like *nos* and *pgc* have no role in this process. In *pgc* and *nos* mutants, PGCs are formed normally, yet specification is at least partially disrupted—and these problems all arise after PGC formation and manifest themselves in different ways during the syncytial and cellular blastoderm stages as well as thereafter (e.g., abnormal migration). Despite the lag between the completion of PGC cellularization and the appearance of various abnormal phenotypes, the functioning of *pgc* and *nos* is not commonly thought to be in “maintenance” but rather in “specification.” If this label is appropriate for *pgc* and *nos*, then it ought to be appropriate for BMP signaling if disruptions in BMP signaling in the period immediately after PGC formation impact processes that are also dependent on these two localized factors.

This scenario is clearly the case. For example, in *pgc* null mutant embryos, the *tll* gene is inappropriately expressed in PGCs, while Pol II CTD phosphoSer2 accumulates in PGC nuclei. These same phenotypes (though less severe: see below) are also observed in the same time frame when *dpp* signaling is partially compromised by viable mutations in *dpp* and *tkv*, or by the removal of *tkv* in the mother. Likewise, in *nos* mutants, *Sxl-Pe* is turned on in PGCs. The same is true when *dpp* is partially compromised.

For these reasons, we think it would be very odd to propose in our manuscript that BMP signaling is required for “maintenance of PGC fate”, while *pgc* and *nos* function during “specification.”

In previous studies, we examined the effects of disrupting BMP signaling during the period when PGCs are migrating to the somatic gonad. In this case, the function would appropriately be labeled “maintenance” rather than “specification”. At this stage of development, *pgc* is likely unnecessary, while *nos* is still required. However, in this case, *nos’s* role in the period spanning migration and gonad coalescence could be labeled as “maintenance’ rather than “specification.”

The question of when and where *dpp* is expressed as well as the issue of “specification” is dealt with further below in responses to points 3 and 4 and also in the response to points made by referee #3.

2) The weakness of the phenotypes raises doubt about the importance of the mechanism invoked by the authors.

The “weakness” of the phenotypes can be attributed to the fact that the mutants used in our experiments are only partial loss of function. Both *dpp^hr92^* and *tkv^427^* are homozygous viable alleles, while stronger loss-of-function alleles are homozygous lethal. The RNAi knockdown is also typically incomplete. We used these alleles (or RNAi KD conditions) because analysis of null alleles (*dpp*) or maternal/zygotic nulls (*tkv*) in the period between nuclear migration into the pole plasm and cellular blastoderm formation is complicated by the difficulties in identifying the embryos that are homozygous mutant at this stage of development. Because of the difficulty in identifying homozygous mutants, we were only able to examine Vasa accumulation in PGCs in embryos homozygous for a hypomorphic but lethal *dpp* allele, *dpp^4^* . *dpp* is haploinsufficient and thus null alleles are very difficult to work with, especially if one wants to genotype blastoderm stage embryos.

3) The absence of a clear topographical relation between the pattern of Dpp expression and the location of pole cells raises he question of whether Dpp is a physiologically relevant germ cell factor during early embryogenesis.

Previously published studies have shown that *dpp* mRNA is expressed on the dorsal side of blastoderm stage embryos, and that expression encompasses both the anterior and posterior pole. At the posterior all of the PGCs are included in the posterior *dpp* expression domain. This is shown in the in situ in Fig. 2A from O’Connor *et al.* 2006 (right). Essentially the same pattern is observed for Dpp-HA (Shimmi et al. 2005).

Consistent with the inclusion of PGCs within the *dpp* expression domain, Dorfman and Shilo (2001) showed that pMad accumulates to high levels in all PGC nuclei. This is shown in Fig 1B of their paper and included here (below, left). There are several other figures in their paper that clearly document pMad accumulation in newly formed PGCs as well. We’ve duplicated their results, and this is shown in the two images on the right below. The most striking finding in Dorfman and Shilo with respect to pMad accumulation in PGCs is that removal of either the maternal *tkv* or zygotic *tkv* gene has minimal effects on pMad accumulation in PGC nuclei. pMad accumulation in PGCs is only lost in the *tkv^M-Z-^* embryos (Fig. 4).

We also confirmed the findings shown in the image from O’Connor *et al.* using smFISH. Below is a syncytial blastoderm embryo probed for *dpp* mRNA. As observed in previous studies, the *dpp* expression domain at the posterior (caret) encompasses the PGCs (highlighted with asterisk).

4) There is a lack of mechanistic insight; no molecular mechanism is proposed as to how downstream component of Dpp signalling feed into PGC fate maintenance.

The key message of our paper is that PGC specification in early embryos is not engineered solely by the maternally deposited and posteriorly localized determinants, but it also depends on inductive signaling mediated by Dpp. This itself is a novel and important discovery, as *Drosophila* has been the classic and by far the best studied example of an exclusively predetermination mechanism of PGC specification. Our experiments demonstrate that this long held view is incorrect and that PGC specification in flies also depends upon the same signaling pathway operating in mammals and other species. It also introduces a new model system for studying epigenesis. As for targets, the PGC phenotypes observed when the BMP pathway is partially compromised point to a close linkage to functions of the three known maternal determinants: *gcl*, *pgc* and *nos*. For each of these determinants, compromising BMP signaling disrupts one or more of their known functions. Whether this is direct or indirect remains to be established and will be the subject of future studies.

5) As performed, the manipulation of Dpp signalling (admittedly difficult at this stage) lack precision in that they do not solely target germ cells or somatic cells. Specifically, RNAi againt Tkv target both pole cells and somatic cells. Definite proof that Dpp signal transduction is specifically required in pole cells would necessitate the transplantation of tkv mutant cells in an otherwise tkv wt embryo (doable but probably challenging for students educated in the modern era).

We agree with the referee that this would be ideal experiment to do—as it would conclusively demonstrate that reception of the BMP signal in PGCs is required proper PGC specification. However, the proposed experiment would have been difficult (impossible) in an era when PGC transplantations were widely used. According to Dorfman and Shilo, pMad accumulation in PGC nuclei is only eliminated when there is no maternal or zygotic source of *tkv*. Therefore, one would have to transplant *tkv^M-Z-^* PGCs into wild type embryos and then determine whether they exhibit the same phenotypes in pre-cellular blastoderm embryos as those that we have found in other sorts of genetic backgrounds. One obvious problem would be identifying which of the donor PGCs have the correct genotype while another would be the difficulties in collecting sufficient numbers of appropriately staged embryos (presumably very soon after each injection) for subsequent experimental manipulations. An equally serious problem would be ruling out side effects from isolating and injecting PGCs. For these reasons the classical PGC transplantation experiments referred to by the referee typically analyzed phenotypic effects in adults, not in blastoderm stage embryos. And usually only a limited number of flies had germlines with the correct genotype (or were fertile).

With respect to “targeting” somatic cells or germ cells: *dpp* is expressed in the soma, not in the PGCs. Thus, when *dpp* function is compromised, any phenotypic defects observed in PGCs arise from a somatic–not a germline–problem. While this means that something in the soma must be important for PGC development, one might argue (given the limitations of our experiments) that there is some novel somatic signaling pathway “X” that is downstream of *dpp:tkv* that is responsible for communications between the soma and germline. While we cannot rule out a two-step soma-germline communication pathway, we think that it would be rather odd to postulate the existence of some novel intervening somatic signaling pathway “X” (activated by Dpp signaling to Tkv in somatic cells) given: a) the high levels of pMad that accumulate in newly formed WT PGCs, and b) the fact that maternal and zygotic sources of *tkv* must be removed in order to eliminate pMad accumulation in PGCs. While these findings don’t exclude existence of “X”, they certainly render this possibility unlikely or implausible.

Overall, this paper makes an interesting suggestion that PGC fate specification/maintenance could be modulated by non-cell autonomous mechanism in Drosophila. However, because of logical inconsistencies and lack of mechanistic understanding, this paper fails to make a convincing case (at least so far). It may be that I missed something in the logical flow and I would be happy to be proven wrong but this would require a much improved manuscript.

Additional comment.

I am a little confused by the experiment illustrated in Fig 6, which shows that pole plasm components are delocalised when Dpp signaling is compromised. This seems inconsistent with the known timing of Dpp expression and pole cell formation. My understanding is that pole cells have formed (and therefore enclosed germplasm) before the onset of Dpp expression. How then could loss of Dpp lead to the release of pole plasm components? Again, I may have missed something but the authors do not make it easy to the reader, especially by specifying a 0-4 hrs stage in the legend (even though precise staging is relatively easy in early Drosophila embryos).

As described above, previously published studies showed that the *dpp* expression domain in syncytial blastoderm stage embryos includes all of the newly formed PGCs. Likewise, all of the newly formed PGCs have pMad in their nuclei, indicating that the ligand is able to activate the canonical signaling pathway in these germ cells. Thus, the phenotypes observed in pre-cellular blastoderm PGCs when components of the *dpp* signaling pathway are compromised can be appropriately attributed to the action (either direct or through “X”) of this somatic signaling pathway.

Here, the referee is asking about the phenotypes (incomplete sequestration of pole plasm) that arise earlier, just before the PGCs complete the process of cellularization. The main negative regulator of *dpp* is nuclear Dorsal. In stage NC9-10 embryos the levels of Dorsal in ventral and lateral nuclei is quite low. Thus, if *dpp* is expressed in NC9-10 embryos one would expect the expression domain would extend if anything even farther ventrally (especially at the poles) than it does in somewhat later cycles. This expectation seems to hold. The images below show that *dpp* mRNAs (red) surround budding PGCs (identified using *pgc* probes) in a WT embryo.

**Referee #2:** This is a beautifully crafted and conceptualized study of germ cell specification that is an important new milestone in our understanding of the germ line development. My suggestions are minor. Strong and convincing evidence is provided to show that Dpp has a critical role at syncytial blastoderm stages and mention is made that Dpp is also important at later embryo stages as well, but no explicit statement or model is offered to suggest where the Dpp sources might be. Perhaps the authors could describe their thinking? My other comments are the small details described in the specific suggestions that follow.

We thank the referee for their generous endorsement. As described in detail in our response to referee #1, above, the Dpp expression domain in pre-cellular blastoderm and cellular blastoderm embryos includes the entire posterior pole of the embryo (and also the anterior pole) and encompasses the PGCs.

We have edited the text to clarify and correct errors, as suggested. Please see new line references following each comment for where the corrected text can be found in the revised version of the manuscript.

Abstract

Ln23 Confusing as not clear if “deployed refers to in a particular animal or, as apparently intended, in biology.

We have tried to clarify this by altering the wording (line 23).

Ln 24 is a signal is a pathway

Corrected on lines 24-25.

Ln 28 not clear if “longstanding model” refers to epigenesis in mammals or preformation in Drosophila

The earlier sentence describes the *Drosophila* model and thus it should be clear that we are referring to preformation.

Author Summary

Ln 37 “to insulate newly formed PGCs from the adverse effects of the cell-cell signaling pathways” - is this an assumption or is it really proven to be the one and only reason for attenuating the cell cycle?

Based on the published data, it would be reasonable to assume that both transcriptional quiescence and mitotic silencing prevent the PGCs from responding to the signaling pathways in the vicinity, which is essential to properly specify and maintain their fate and their cell cycle. For instance, mitotic cell cycle of PGCs is known to be regulated by germ cell autonomous regulators Nos and Pum.

Introduction

Ln 63 what is a “somatic” gene?

Somatic genes are those that are specifically required for somatic patterning and are not active in the germline.

Ln 65 Because all the lineages are fixed in worms, is it correct to distinguish one product of the first division as “set aside”?

In worms, germline soma segregation happens during early cell divisions where P4 is designated to serve as a germ cell precursor.

Ln 69 What cycle is this minor wave of ZGA?

The minor wave of ZGA begins at nuclear cycle NC8 (line 70).

Ln 71 What cycle is this major wave of ZGA?

The major wave of ZGA starts at nuclear cycle NC14 (line 71).

Ln 85 Not clear what the new term “model invertebrates” refers to – flies and worms? Does “higher animals” refer to mouse and human, or to other vertebrates as well?

We have specified this now on lines 86-87.

Results

Ln 172 The described result implies that twi-Gal4 is expressed and effects expression of UAS targets at stage 5/6. It would be reassuring to examine expression of a UAS target directly, perhaps UAS-GFP?

Fig 1 Please show twiGal4 expression at the stages analyzed for Cyclin B, pH3, and PGC number.

We confirmed that *twist-Gal4* is expressed in a consistent manner using *UAS-βgalactosidase*. The anti-βgalactosidase antibody staining of the embryos was observed from late stage 4/5 onwards and faithfully reproduced the anti-Twist antibody staining pattern in young embryos, which is localized on the ventral side and thus complements *dpp* expression.

Ln 216 Perhaps change “significantly” to “detectably”?

Corrected on line 218.

Ln 276 Change “embryos” to “embryo”

Corrected on line 278.

Ln 408 Please explain why dpERK was monitored

The doubly phosphorylated form of ERK kinase is used as a diagnostic marker of activation of the pathway. This is now included in the text (line 413).

Ln 513 Adhesion is assumed, not demonstrated by these observations of distribution/localization

Indeed changes in adhesion is logical and likely explanation but it is not proven.

Corrected on line 527.

M&M

Ln 652 Michael O’Connor mis-spelled

Corrected on line 682.

**Referee #3:** Across the animal kingdom two mechanisms of primordial germ cell (PGC) specification have been identified, which the authors call here preformation and epigenesis. In Mus musculus, extrinsic BMP signals induce PGC specification, while in Drosophila melanogaster and Caenorhabditis elegans, maternally deposited determinants were thought to be necessary and sufficient to establish PGCs. Following up on prior work from their group that showed that PGCs are sensitive to BMP signalling after initial specification, in this manuscript, the authors attempt to show that BMP signalling, a known regulator of PGC specification by epigenesis, is also required during the early embryonic process of D. melanogaster germline specification. The manuscript is very well written, easily accessible and the data are for the most part well presented. Weaknesses include several unclear experimental design choices, ambiguity in methods, and most importantly, a lack of clear and

consistent definition of what the authors consider are the differences between crucial cell fate concepts including “specification,” “formation,” “differentiation,” and “maintenance.” In its present form, I believe the manuscript will leave most readers familiar with the field wondering why the authors are claiming to overturn a long-standing understanding of Drosophila PGC specification, rather than claiming to provide additional evidence that PGCs use BMP signaling for some aspects of their differentiation, whose ultimate impacts on the ability of PGCs to function as such are not explored.

Major comments

1. The crux of this manuscript - the term that readers are likely to focus on, and that the authors build their major interpretation around - is the term “specification”. The authors begin by defining the hallmarks of specification to include the suppression of transcription and the recruitment and maintenance of germplasm. While they show that a change in BMP signalling does affect some molecular markers of both processes, these cells continue, to some extent, to express Vasa, which is a key constituent of a PGC. The authors do not address whether the PGCs that show aberrant molecular markers of various processes, are still able to function as PGCs or not. Thus, if the definition of “specified” means “able to fulfill a specific biological function,” these experiments do not address that. If instead, “specified” here means “displaying specific molecular markers to a specific degree at a specific developmental time point,” then this is more precisely what the experiments

address. The manuscript does not look at the expression of oskar which is, as they explain, the major autonomous regulator (Line 112) of PGC specification. The authors also confuse their own argument by referring to cells which do not express Vasa or continue to express tll as PGCs, which to this reviewer and I believe, to many readers, means that they are not specified as PGCs. The early localisation of germ plasm determinants at the posterior of the embryo, followed by its dispersion, is indeed evidence for the role of BMP signalling in the maintenance of germ plasm localisation by downregulating the posterior cascade. This is a very interesting role for the BMP signalling pathway, but without a very clear definition of how the authors are using the term “specify,” does not clearly amount to “specification” of the PGCs in the mammalian sense as the authors imply.

Here referee #3 raises similar questions about terminology that were mentioned by the other referees. As indicated in our response to referee #1, *gcl*, *pgc*, and *nos* are commonly thought to be *osk*-dependent, germ cell autonomous factors that function in the *specification* of PGCs. Each gene has unique and overlapping functions in the specification process, and mutations in these three genes differentially affect PGC specification. *nos* mutants have no effect on PGC formation; however, they disrupt the specification process and (when rescued for patterning defects by simultaneous maternal *hb* loss) fail to develop a functional germline. *gcl* and *pgc* mutants also disrupt the specification process, but the effects of mutations in these two genes is not complete (perhaps in part because of their overlapping functions). In both instances a functional germline is formed in apparent null alleles. Thus, these two genes would be considered to encode “important” but not “essential” specification factors. As discussed in our response to referee #1, if these three genes are considered to be “specification factors”, then any other gene that encodes either a maternal determinant or a zygotically active factor that when compromised/mutated exhibits a similar set of PGC phenotypes in the same time frame would logically be considered to have some role in the specification process. As with *gcl* and *pgc*, such genes could be important but not essential and still function in specification. They could also be essential.

While the experiments in our paper do not demonstrate that the BMP pathway is *essential* for PGC specification, they clearly show that it is *important* for proper specification of PGC identity in early embryos.

As noted above, many of the phenotypes evident in *gcl*, *pgc* and *nos* mutants are also observed when *dpp* or *tkv* activity is *partially* compromised. These phenotypes include a) failure to properly sequester germline determinants, b) reduced levels of the PGC marker Vasa in a subset of PGCs, c) inappropriate activation of the terminal pathway, d) failure to properly downregulate transcription and e) precocious invasive migration of PGCs All of these phenotypes are consistent with defects in the proper specification of PGC fate. Moreover, there is a synergistic genetic interaction between the maternal germline determinant *osk* and *dpp* that gives rise to a similar set of phenotypes as those observed in *gcl*, *pgc*, *nos*, *dpp* and *tkv*. In this genetic interaction, the dose of *osk* is reduced by half in the mother, while the offspring have a WT *dpp* allele *trans* to a homozygous viable *dpp* allele. That specification phenotypes reminiscent of those observed for localized germline determinants in this weakly sensitized genetic background indicates that BMP function in the early zygote is closely linked to that of the germline determinants whose localization depends upon *osk*.

As to whether *“*BMP compromised” PGCs can fulfil their normal functions: We have examined the effects of compromising BMP signaling to PGCs (by RNAi knockdown of *tkv* in PGCs using a *nos* driver) during mid-embryogenesis in the period preceding gonad coalescence. As is the case in pre-cellular blastoderm embryos, we observe a variety of PGC phenotypes. These include the loss the PGC marker Vasa and the GSC marker (which appears before gonad coalescence is complete) spectrosome. These findings would be consistent with the idea that continuous BMP signaling is likely to be required for PGCs to successfully fulfill their normal functions—namely to coalesce with the embryonic gonad and assume a GSC identity. Also note that in these older experiments, as well as in the experiments presented in this manuscript, we were only able to *partially* compromise the functions of the BMP signaling factors.

In this context, it is useful to revisit the evidence that localized maternal determinants are solely responsible for PGC specification in flies. In these experiments, PGCs are formed either by transplantation of pole plasm or by *osk* mislocalization, and their function is then assayed by their ability to form a functional germline. In all of these experiments, the test PGCs are in the same environment as normal PGCs and thus are receiving and responding to the Dpp ligand (Dorfman and Shilo, 1999). There is no evidence that PGCs specified solely by maternal determinants (e.g pole plasm placed on the ventral side of the embryo) and then allowed to develop in the complete absence of BMP signaling would be able to form a functional germline. Given the deleterious effects of limiting BMP signaling that are evident not only in pre-cellular blastoderm embryos but also later in embryogenesis prior to gonad coalescence, it would be reasonable to think that specification would fail.

While flies clearly differ compared to mammals in that they deploy localized determinants, the finding that BMP signaling is also required for specification clearly represents a relevant similarity.

Referee #3 also asks why we didn’t look at *osk* localization in the zygote. As far as we know, the key functions of *osk* are maternal, not zygotic. Those functions—assembly of the pole plasm during late oogenesis—should not be impacted by a disruption of the BMP signaling pathway in the zygote. Consistent with this idea, the association of the pole plasm with the posterior cortex in pre-NC9/10 embryos compromised for BMP signaling pathway is unaltered (S6 Fig) Only after the nuclei enter the pole plasm do we observe alterations compared to wild type. So even if we discovered that *osk* mRNA (and protein) were mislocalized when BMP signaling is perturbed, one would have to imagine novel zygotic functions in order to assign any significance to such a finding. However, in the absence of any known activity in the zygote that is critical for PGC specification, this experiment does not seem to be especially important at this juncture.

It is also worth noting that *osk* mRNA is not a constituent of germ granules that contain mRNAs required for PGC development. *osk* mRNA is associated with the founder granules (a new kind of RNP granules identified by the Gavis lab) which are distinct from the germ granules. They also appear to be largely excluded from the PGCs rather than being specifically incorporated into the PGCs.

2. “Embryos” and “PGCs” are described interchangeably as having “reduced expression” in the paragraph starting with line 226. This is part of a larger issue, which is that the authors’ logic in sometimes quantifying the number of PGCs with a specific phenotype, and in other experiments, quantifying the number of embryos containing any PGCs at all with a specific phenotype, is unclear. For example, the authors show an increase in tll transcript in a few cells per embryo in Figure 4, and at this point in the manuscript begin to represent the data as the number of embryos which have any PGCs that show a change in tll expression levels. The same method is repeated for slam and Sxl expression (Figure 6 and Table S6), and the reason for changing their methodology for these experiments is unclear. They need to state explicitly why they do so, and if they wish to continue using the “number of embryos” approach then they need to specify how many cells within each of these embryos

show such a phenotype.

We present transcription data for *tll*, *slam* and *Sxl-Pe.* While we utilize traditional, antibody-based FISH to examine the expression pattern of *tll*, we analyzed *slam* and *Sxl-Pe* expression using smFISH. The text presents the key quantification in each case. For *tll* we find that 70% of the *dpp^hr92^* embryos have *tll* mRNAs in their PGCs compared to 0% for the WT control. For *slam,* we find that 26% of the *dpp^hr92^* embryos have *slam* mRNAs in their PGCs while 35% of the *dppi^33618^* embryos have *slam* mRNAs. Finally in the case of *Sxl-Pe* 18% of the *dpp^hr92^* embryos have *Sxl-Pe* transcripts. Because the *Sxl-Pe* probe is homologous to intron sequences, we only detect nascent transcripts. Note that even in the soma, transcriptional bursting for *Sxl-Pe* (and other genes) is stochastic and not all nuclei give two signals in female embryos. Finally because *tll* mRNAs are uniformly distributed through the cytoplasm (as compared to *slam* which is membrane localized) and traditional FISH was used to detect mRNA we were also able to quantitate the “relative levels” of *tll* mRNA in mutant and WT PGCs.

3. Many of the phenotypes examined are quantified, but a notable exception to this is Vasa protein levels, which are described only qualitatively as “high” or “low” without explaining how a reproducible threshold was established for this scoring (e.g. Figure 2 and Figure 4). It is also unclear whether the authors counted all PGCs in each embryo, or a subset of PGCs from each embryo or each biological replicate, and if the latter, how they selected that subset. The same is true of Cyclin B levels in Figure 1.

The differences in Vasa protein levels were consistent and PGCs could be easily classified. The individual PGCs were counted using both projections and single slices. PGCs were classified into low Vasa and high Vasa categories, and it was confirmed by doing a ‘double blind’ counting.

If a PGC had high levels of Vasa (or even close to WT levels), it was considered WT. Slight reductions were not counted, nor were reductions that were only obvious in single slices. PGCs were counted by going through each slice of a Z stack and categorizing each cell (viewed throughout all relevant slices) as WT (high) or low levels of Vasa.

In the case of both Vasa and Cyclin B (and any other PGC counts throughout the manuscript), all PGCs of each embryo were counted.

4. To rule out the possibility that experiments are detecting increased or decreased PGC numbers relative to controls, because of discrepancies in the developmental stages of embryos scored for controls and experimental genotypes, the authors should provide more details in the Methods to clarify how they were certain that all scored embryos were at precisely the dame developmental stage.

While counting PGCs, we ensured that differences were not due to different cycles or stage. To compare, the same number of embryos at identical nuclear cycles were counted between the experimental and control categories. Also, the differences were reproducible between the trials and confirmed using pH3 as well as CyclinB staining.

Minor comments

1. Please consistently refer to all tables and figures be referred to as Table X or Figure X throughout the manuscript. There are many variants across the text and between the main figures and the supplementary figures. The supplementary figures are called “X figures,” for example.

We have double-checked to ensure that we are referring to the correct figures and tables in the text. All references to figures and tables are according to the style of PLOS journals, so we have not altered mentions of “S1 Fig”, etc. All main figures/tables are to be referred to with the number following the classification of figure/table while supplemental material is referred to as S[number] followed by the classification of figure/table.

2. Throughout the manuscript and in all figure legends, please include complete genotypes of all samples, rather than abbreviations. This includes all instances of “WT” and “control.” Please also clarify which experiments use germline clones and which are zygotic mutants, as it is not clear in a few instances (e.g. mentioned in line 245 rather than line 229).

For the embryo staining, we used *white^1^* embryos as a control in most instances. Wherever the experiment involved an RNAi knockdown strategy, we generated *egfpi* embryos by crossing *maternal-tubulin-GAL4* virgins with *UAS*-*egfpi* males and used these as control.

This is mentioned in the first section of ‘Materials and Methods’ (lines 684-686).

3. In Figure S1, the UAS:dpp experiment generates ectopic expression and not a gain of function – please correct this. Though the legend for this figure states that Vasa is being used to assess pole cell proliferation, it is unclear how that is being measured. Finally, C and D are supposed to be comparable to each other, but it is unclear whether the images shown are of the same region of the embryo - if so, then there appear to be many more than the 5 extra PGCs described in the text (Line 142) and in Table S1.

As mentioned previously, PGC counts were made using equal number of embryos at same stage between different genotypes. While modest, the increase in PGC number due to excess Dpp in the vicinity was reproducible and was conformed using two independent Gal4 drivers.

4. On line 179, though 2.8 is technically more than double of 0.7, it may be more accurate to say “quadruples” or “four-fold increase”.

We have changed this to ‘considerably elevated’ and mentioned an approximate four-fold increase (line 186).

5. On line 203, the data showing the relative nos levels between the WT condition and the Dpp over-expression condition are not shown. These data need to be shown to make the claim in the earlier sentence. Another option would be to remove these two sentences from the manuscript.

We have removed these sentences, as suggested.

6. On line 226 the authors should rephase the sentence “As expected from previous studies, we found that the level of pMad in the PGCs of dpphr92 and tkv427 embryos is reduced compared to WT [52].” The authors do not show expression of pMad in these mutant conditions.

Thank you for the suggestion. We have now rephrased this sentence to clarify (lines 228-229).

7. In Figure S2, though there is a clear increase in Vasa expression, it is unclear how the asterisk indicates excess cell division.

We have fixed the text as suggested.

8. Figures S3 and S5 are never mentioned in the text of the manuscript.

Thank you for catching this error. We have now added appropriate references to the supplemental figures in the manuscript (lines 283 and 367, respectively).

9. The values in line 235 do not match the data in Table S4.

Thank you for pointing out this mismatch. We have now corrected the typo so that the text (line 236) matches the data (which has the correct numbers) in S4 Table.

10. The values in line 281 do not match the data in Table S5. In addition, the sum of the first two columns in each row, does not match the sum of the final two columns. This suggests that not all PGCs were counted for all embryos scored, but this point is unclear in the manuscript.

Thank you for pointing out this mismatch. We have now corrected the typo so that the text (line 283) matches the data (which has the correct numbers) in S5 Table. Furthermore, we have added columns in S5 Table to clarify how many PGCs were counted to assess pSer2 and H3meK4 in PGCs, which were scored in independent experiments.

11. In the legend for Figure 4, G and G’ are not mentioned or described.

We have now added a reference to G and G’ panels in the legend of Figure 4.

12. The figure legend for Figure 5 does not accurately represent the data. B is mentioned with two different genotypes.

We have corrected the legend for Figure 5 to refer to the correct genotypes.

13. In Figure 6D, the nomenclature of the genotypes (e.g. dpp allele name) varies from the rest of the manuscript. What is “egfi”?

Explained as suggested. The genotypes have been mentioned earlier in the text, also.

14. All references to Pae et al. are missing a date (lines 401 and 619).

Per the reviewer’s suggestion, we have now mentioned the year in which Pae *et al.* published the study (lines 405 and 638, respectively). Numerical references to Pae *et al.* 2017 are also included in these sentences.

15. The information provided in lines 128-131 and lines 134-137 is redundant and should be combined.

It seems to us that the info in this section of the intro highlights the ability of PGCs to respond to extracellular signals even before they coalesce with somatic components of the gonad. It also indicates that the response to different signaling molecules is not necessarily the same as that engendered in somatic cells.

16. In the Materials & Methods, please provide a more detailed explanation of how exactly the Opo SOS was activated in the embryo.

A description with more details has been included in ‘Materials and Methods’.

17. Please replace descriptions of germ plasm components “spreading”:into somatic cells, which implies an active movement that the authors did not observe, with a more neutral description of the locations where they observed the germ plasm components that they assessed, at different developmental time points.

We have changed these descriptions as recommended throughout.

18. Figure 9 – please add an appropriate control genotype to the spatial quantification of pgc levels, and to Table S2. For example, dpp[hr92]/+.

Indeed, the referee is correct in pointing out that this control has not been included. We relied on the observation that homozygous *dpp^hr92^* embryos did not show appreciable invasive migration phenotype (see newly added S7 Table) or other changes in PGC morphology or behavior. Thus, *dpp^hr92^*/+ embryos are likely to resemble wild type and thus were not analyzed.

19. Lines 522-533 – why is there no quantification of the phenotype for these two genotypes, when such quantification is provided for all other phenotypes described in this paragraph? Please provide quantification for all genotypes.

We have fixed this, as suggested. These data are included in S7 Table.

20. Table S8 – please indicate how many embryos total were scored for each genotype, and provide a control genotype.

We have now split Table S8 into two tables for clarity (and tables have been renumbered as we have included more data in the revised version). Table S9 now contains the counts of embryos scored for Vasa while Table S10 contains data for embryos scored for PGC behavior, as well as the total embryos assessed for particular phenotypes.
