# Supplementary material for: Preformation and epigenesis converge to specify primordial germ cell fate in the early Drosophila embryo
Source: PLoS Genet. 2022 Jan 5;18(1):e1010002. doi: 10.1371/journal.pgen.1010002 (PMC8765614; doi:10.1371/journal.pgen.1010002)
Supplement: Attachment — Submitted filename: Colonnetta et al._PLOS Genetics Response2_Final.docx [file pgen.1010002.s021.docx]

Gregory P. Copenhaver
Editor-in-Chief
PLOS Genetics

Dear Dr. Copenhaver,

Dear Dr. Bosco,

Thank you for the reviews of our manuscript (PGENETICS-D-21-01030R1: Preformation and epigenesis converge to specify primordial germ cell fate in the early *Drosophila* embryo).

In our reading of the reviews, referee #2 appeared to be satisfied and in favor of publication.

Referee #1 wondered why we did not use the strong loss of function conditions for both *dpp* ligand and the *tkv* receptor. Had this been technically feasible, we would have done so. Null alleles of *dpp* are haploinsufficient and must be carried over balancers that have a *dpp* duplication. We did use a strong loss of function allele, *dpp^4^*, to examine Vasa protein accumulation in PGCs. *dpp^4^* was recombined with a *twist* protein null mutation. Staining for Twist protein allowed us to identify *dpp^4^* embryos. However, because *twist* expression is only activated several nuclear cycles after PGCs are formed, we could not use this marker to identify homozygous mutants during the earlier steps in PGC specification, which is the period of most interest. Precisely the same problem would arise had we followed the referee’s suggestion and used the tagged balancers (*twist-gfp* or *eve-lacZ*). As for *tkv*, previous studies by Dorfman and Shilo indicated that *tkv* must be mutant in mother and in the zygote to prevent pMAD accumulation in PGCs. Again, there is a problem identifying *tkv^m-z-^* embryos during the early steps in PGC specification. However, we would like to point out that for the *tkv* receptor, we employed a null allele of *tkv* (*FRT-tkv^8^*) to generate the germline clones. The embryos derived from females carrying *tkv* germline clones displayed loss of Vasa in young PGCs and the ectopic presence of transcriptional marker (phospho-isoform of RNApolII or anti-Pser2).

Based on our observations, we feel that complete loss of *dpp* will likely result in more severe and penetrant PGC specific phenotypes (extensive loss of Vasa, penetrant ectopic transcription of somatic genes, changes in chromatin architecture etc.). Moreover, such analysis may also uncover an earlier and/or novel function of Dpp pathway during precocious cellularization of pole buds or during anchoring of RNA to the posterior cortex in presyncytial embryos. As suggested, we have included a short discussion of these possibilities in the revised version of the manuscript.

As for the functional significance—it is clear that compromising BMP signaling impacts the process of PGC specification, giving phenotypes that are the same as those observed in embryos from mothers mutant in the PGC determinants *nos*, *pgc,* and *gcl*. As previously pointed out, these three genes have overlapping functions and at the blastoderm stage do not completely disrupt the process of PGC specification. This is true for the BMP pathway as well.

In closing, we have modified the text to address the concerns raised by the reviewer. Please find a detailed response to the referee’s comment below. We hope that the revised version of the manuscript will be considered suitable for publication in *PLOS Genetics*.

Sincerely,

Girish Deshpande

**Responses to Reviewer Comments:**

Comments to the Authors:

**Reviewer #1:** The main problem for the first draft was that the paper did not address the functional significance of BMP signalling in germ cell specification. Unfortunately, the authors did not add experimental evidence to strengthen this statement in the current version. They attribute the weakness of the phenotype to the hypomorphic dpp and tkv alleles they used, but not explain why they did not use the stronger LOF alleles instead. Moreover, they claim that it is difficult to distinguish homozygous mutant embryos at the blastoderm stage, which limited them from more careful analysis. But my understanding is that this is possible with Cyo Twist-GFP or CyO,eve-lacZ. Moreover, there remain problems with imaging quality - For example, the figures presented in the response letter (on page 11) showing the distribution of dpp RNA is not informative due to extremely high background signals.

**Response:**

In our view, the functional significance of Dpp signaling during PGC specification is clearcut. It is required for the proper specification of PGCs as indicated by its requirement during maintenance of germ cell specific markers such as Vasa, and proper anchoring and/or transmission of germ plasm components such as *gcl* and *pgc* RNA. Consequently, when *dpp* signaling is compromised, PGCs display ectopic transcription of somatic genes, which is reflected in both the presence of individual transcripts (*tll*, *Sxl,* etc.) and general markers such as the presence of pSer2 and H3MeK4.

The reviewer is correct in stating that we did not use null alleles of *dpp* to analyze the phenotypic consequences. We would, however, like to point out that we did use a null allele of *tkv* (*FRT-tkv^8^*) to generate embryos from females carrying the germline clones. These data are reported in the manuscript and indicate that maternal loss of *tkv* mimics the zygotic loss of *dpp*. Thus, it is our impression that complete loss of *dpp* (zygotic)or *tkv* (maternal and zygotic) will likely result in more severe phenotypes. We have summarized some of the possible outcomes in the revised Discussion section (lines 584-590):

“It is also worth noting that the functioning of the BMP pathway was not completely disrupted in our experiments. Thus, the possibility remains open that more dramatic or even some additional phenotypic effects might be observed under conditions where BMP signaling was entirely absent in pre-cellular blastoderm embryos. (For instance, *dpp* activity may affect pole bud formation or PGC cellularization. It will also be of interest to assess if pole plasm anchoring is completely normal in pre-blastoderm embryos if BMP pathway is compromised).”
